# Association of health-risk behaviors with perceived academic performance among middle and high school students: A cross-sectional study in Shanghai, China

**DOI:** 10.1371/journal.pone.0285261

**Published:** 2023-05-02

**Authors:** Chunyan Luo, Xuelai Wang, Yanting Yang, Qiong Yan, Lijing Sun, Dongling Yang

**Affiliations:** Division of Child and Adolescent Health, Shanghai Municipal Center for Disease Control and Prevention, Shanghai, China; Shahid Beheshti University of Medical Sciences, School of Dentistry, ISLAMIC REPUBLIC OF IRAN

## Abstract

Adolescence is a susceptible period to establish health-risk behaviors, which may have an impact on academic performance. The aim of this study was to investigate the association between health-risk behaviors (HRBs) and perceived academic performance (PAP) of adolescents in Shanghai, China. The data of the present study included three-round Shanghai Youth Health-risk Behavior Survey (SYHBS). This cross-sectional survey investigated multiple HRBs of students involved in dietary behaviors, physical activity and sedentary behaviors, intentional and unintentional injury behaviors, and substance abuse behaviors, as well as PAP by using self-reported questionnaire. Using a multistage random sampling method, 40,593 middle and high school students aged 12 to 18 years were involved. Only participants with complete data on HRBs information, academic performance and covariates were included. A total of 35,740 participants were involved in analysis. We used ordinal logistic regression to analyze the association between each HRB and PAP adjusting for sociodemographic, family environment and duration of extracurricular study. The results showed that students who did not eat breakfast or drink milk everyday were more likely to have a lower PAP, with a decreased odds of 0.89 (95%CI: 0.86–0.93, *P*<0.001) and 0.82 (95%CI: 0.79–0.85, *P*<0.001), respectively. The similar association was also found in students who did exercise ≥60 minutes for less than 5 days/week, spend time on watch TV beyond 3 hours/day and other sedentary behaviors. Most intentional and unintentional injuries, and ever smoked were associated with a lower PAP. Our finding suggests that multiple HRBs negatively associated with PAP of adolescents. It needs to raise public health concerns with HRBs in adolescents, and to develop and implement comprehensive interventions on HRBs.

## Introduction

Adolescents, defined by the United Nations as those between the ages of 10 and 19, make up 16% of the world’s population [1]. Physical and mental health issues increased significantly in adolescents over last decade. For example, globally, it is estimated that over 1 in 6 adolescents was overweight, and 1 in 7 adolescents experience mental health conditions [2, 3]. In China, at least 30 million children and adolescents under 17 years of age struggle with emotional or behavioral problems [4]. The prevalence of overweight and obesity among adolescents aged 13–18 has risen dramatically to over 21% in 2019 [5]. Most of health issues during adolescence are preventable or treatable; however, adolescents are generally overlooked in health information, policies and services, around the world [6].

Adolescence is an important time for establishing good faith on health and developing a healthy lifestyle. Meanwhile, adolescence is a sensitive period to form health-risk behaviors (HRBs) [7]. HRBs in adolescents usually includes unhealthy dietary behaviors, inadequate physical activity, intentional and unintentional injury, substance abuse, and sexuality that may lead to unintended pregnancies and sexually transmitted diseases [8]. These behaviors are not only contribute to the leading causes of illness and death in adolescents, but also related to the brain development, particularly for cognitive ability and academic performance [9, 10]. HRBs can further produce long-term effect on health in adulthood [11, 12]. On the other hand, early prevention programs during adolescence are the most cost-effective interventions for investment by countries, [6, 13] which can minimize subsequent disease burden in adulthood, and ultimately affect the health of the next generation [11]. Therefore, it is essential to understand local situation of HRBs among adolescents, and develop targeted interventions.

Academic performance is a topic of concern to adolescents, parents, and teachers around the world. A mounting pressure of academic contributed to a large amount of time on study, which prompted more academic-related sedentary behaviors and other HRBs in students [14]. In China, only 34.1% of school-aged children and adolescents meet moderate-vigorous physical activity (MVPA) guidelines [15]. The development of HRBs, in turn, has a reverse effect on students’ academic performance. A Singapore intervention experiment reported that adolescents who skipped breakfast and remained sedentary over a morning presented lower mathematical task performance and speed [16]. In other words, healthy behaviors have been found to associate with better academic performance. A follow-up study from Canada indicated that moderate-to-high-intensity physical exercise, vegetable and fruit intake during childhood at the individual level could be used as predictors for behaviors in the sample population [17]. There were, however, only a few study concerned with the impact of HRBs on academic performance in Chinese adolescent students [18]. As one of the largest and most developed cities in China, Shanghai provides abundant educational resources for children. The Program for International Student Assessment (PISA) 2018 showed that on average, 15-year-old students in Shanghai (China) outperformed students from other countries in reading, mathematics and science [19]. On the other hand, the surveillance data in recent years indicated that most adolescents in Shanghai were not meeting recommendations for healthy eating, physical activity, or sleep duration [20, 21]. Thus, we should arouse wide concern among schools, families, and students in Shanghai with the impact of HRBs on academic performance through a comprehensive survey.

Surveys of adolescent health behaviors are mainly questionnaire-based [22]. The Youth Risk Behavior Surveillance System (YRBSS) is the largest public health surveillance system in the United States dedicated to monitoring a broad range of HRBs among middle school students [8]. It was developed in 1990 and conducted by the Centers for Disease Control and Prevention. Based on the advanced experience of YRBSS and combined with the situation of China, Shanghai Municipal Center for Disease Control and Prevention (SCDC) has carried out the Shanghai Youth Health-risk Behavior Survey (SYHBS) to monitor HRBs among middle and high school students aged 12 to 18 years in Shanghai since 2004. In present study, three-round data of SYHBS were aggregated for analysis. We aimed to investigate the potential association of each HRB and academic performance among middle and high school students in Shanghai, and to provide evidence for education and health departments to develop early prevention strategies, programs, and interventions for adolescents. If HRBs are related to academic performance, there is important significance for local education and health departments to carry out health promotion regarding HRBs within communities, schools, and families.

## Materials and methods

### Sample and setting

The SYHBS was an anonymous cross-sectional survey of middle and high school students from grade 6 to 12 in Shanghai. It was conducted every two to four years. We adopted multistage random sampling by dividing the schools in each district into two strata, middle school and high school, based on the educational levels. According to the proportion of students in each district of Shanghai, probability proportional to size (PPS) sampling was adopted in each stratum to select schools. Then, the classes were randomly sampled in each grade of the selected schools. All students in the selected classes were required to filled out the questionnaire anonymously.

In this study, we analyzed the data in 2004, 2012, and 2019, including a total of 40,593 middle and high school students. Only participants with complete data on HRBs information, academic performance and covariates were included, with a sample of 35,740 students. The study has been approved by the Ethics Committee of SCDC (2022–1). We got permission from each school. All school principals provided written informed consent before the surveys.

#### Procedures

The SYHBS was initiated by SCDC and implemented by the CDC in 16 districts in Shanghai [20, 23]. Each District Departments of Education supported the launch of surveys in middle and high schools in Shanghai. Survey investigators included school health professionals from SCDC and 16 district level CDCs in Shanghai, who were underwent unified training. The information filled in by the students was kept strictly confidential.

#### Measures

The questionnaire adopted was Shanghai Youth Health-risk Behavior Survey Questionnaire (SYHBSQ), which has a test-retest reliability of 0.93 [24]. SYHBSQ consisted of basic demographic information (age and gender), the HRBs, academic performance, family environment (maternal education and family structure), and duration of extracurricular study.

In current study, the concerned HRBs consisted of four dietary behaviors, four physical activity and sedentary behaviors, four unintentional injuries, nine intentional injuries, and four substance abuse behaviors. The dietary behaviors involved eating breakfast, drinking milk, drinking soda, and eating dessert. The physical activity and sedentary behaviors included the spending time on doing exercise, watching TV, surfing the Internet, and video game. The unintentional injuries referred to jaywalking, cycling violations, swimming in unsafe regions, and severely injured. The intentional injuries included physical fight, feeling unsafe on the way to school, being bullied, feeling lonely, academic stress, insomnia, feeling depressed, and run away from home. Substance abuse behaviors involved tobacco smoking and alcohol drinking. Table 1 presented each HRB description and coding for analysis.

**Table 1:** Definition and assignment of health-risk behaviors.

Health-risk behavior	Variable description	Coding for analysis
**Dietary behaviors**		
Daily breakfast intake	Number of days having breakfast in the past 7 days	0: 7 days; 1: <7 days
Daily milk intake	Number of days consuming milk, yogurt, or soy milk for in the past 7 days	0: 7 days;1: < 7 days
Drinking soda	Frequency of soda drinking in the past 7 days	0: <1time/day; 1: ≥1 time/day
Eating dessert	Frequency of eating dessert in the past 7 days	0: <1time/day; 1: ≥ 1 time/day
**Physical activities and sedentary behaviors**		
Exercise	Days of exercises for at least 60 minutes in the past 7 days	0: ≥ 5 days; 1: <5 days
TV watching	Time spent watching TV or videos per day for the past 7 days	0: <3 hours/day; 1: ≥3 hours/day
Duration of Internet-surfing	Time spent on Internet-surfing in the past 7 days	0: <3 hours/day; 1:≥3 hours/day
Video gaming	Time spent on video games per day in the past 7 days	0: <3 hours/day; 1:≥3 hours/day
**Unintentional injuries**		
Jaywalking	Walking across the street without crossing at the crosswalk/pedestrian bridge/underground passage in the past 30 days	0: No; 1: Yes
Cycling violations	Including cycling chasing, traveling on the wrong direction, running a red light, jay biking, giving rides to passengers on a bike	0: No; 1: Yes
Swimming in unsafe regions	Swimming in places with no safety measures in the past 12 months	0: No; 1: Yes
Severely injured	Severely injured	0: No; 1: Yes
**Intentional injuries**		
Physical fight	Physical fight with someone in the past 12 months	0: No; 1: Yes
Feeling unsafe on the way to and from school	Feeling unsafe on the way to and from school in the past 12 months	0: No; 1: Yes
Being bullied	Including malicious teasing, demanding property, isolation, threatening, kicking, and locking up, dirty jokes	0: No; 1: Yes
Feeling lonely	Feeling lonely in the past 12 months	0: No; 1: Yes
Academic stress	Unhappy mood due to academic stress in the past 12 months	0: No; 1: Yes
Insomnia	Loss of sleep in the past 12 months due to worrying about something	0: No; 1: Yes
Feeling depressed	Feeling saddened for 2 consecutive weeks or longer to stop usual activities (depression tendency)	0: No; 1: Yes
Runaway thoughts	Thinking about running away from home	0: No; 1: Yes
Runaway behavior	Have run away from home	0: No; 1: Yes
**Substance abuse behaviors**		
Ever smoked	Have tried smoking	0: No; 1: Yes
Currently smoked		0: No; 1: Yes
Ever alcohol drank	Have tried alcohol	0: No; 1: Yes
Currently alcohol drank		0: No; 1: Yes

Perceived Academic Performance (PAP) was assessed by a single-item measure, “How would you rate your overall academic performance compared with classmates?”. Students could select one of the following response options: poor, below average, average, above average, good, and not sure.

We selected covariates which were potentially related to HRBs and academic performance. Covariates included student’s gender (male or female), maternal education (elementary school and below, middle school, high school, junior college; bachelor’s degree and above), family structure type (nuclear, non-nuclear), extracurricular study time for the past 7 days (<4 h/day, ≥4 h/day).

### Data analysis

We entered data with Epidata 3.1 and analyzed data by SPSS 25.0 software package (IBM, Armonk, NY). The distribution of each HRB among study population was present with a composition ratio (%). The Chi-square test was used to compare the distribution of PAP with different sociodemographic characteristics. Finally, we performed ordinal logistic regression to examine the association between each HRB and PAP in crude model and adjusted model, respectively. A *P* value < 0.05 was statistical significance.

## Results

### Sample characteristics

Table 2 describes the population characteristics and response rates in 2004, 2012, and 2019, respectively. The number of respondents in each survey in this study was the same size calculated according to the sampling method and the behavior prevalence. The sample sizes of the individual surveys in the three years were 8,597, 18,820, and 13,176, respectively accounted for 1.03%, 3.21%, and 2.36% of the total number of middle and high students in Shanghai. The response rates for the three surveys were 92.22%, 92.24%, and 97.75%, respectively.

**Table 2:** Population distribution and responses of SYHBS in 2004, 2012, and 2019.

	2004	2012	2019
Number of schools participating in the survey	76	58	54
Total number of middle and high schools	866	763	875
Number of students participating in the survey	8597	18820	13176
Total number of middle and high school students	834519	587191	557318
School response rate	100.00%	100.00%	100.00%
Student response rate	84.44%	84.47%	95.50%
Overall response rate	92.22%	92.24%	97.75%

The average age of the students in this study was 14.9±1.9. The distribution of students with poor PAP, below average PAP, average PAP, above average PAP, and good PAP were found in a total of 2,940 (8.23%), 6,856 (19.18%), 11,060 (30.95%), 9,984 (27.94%), and 4,900 (13.71%), respectively. As shown in Table 3, significant differences in age, gender, school type, family structure, maternal education, extracurricular study time, and survey year were found in students with different PAP. Table 4 reports the prevalence of each HRB among adolescents from the lowest rate of currently smoking (2.4%) to the highest rate of academic stress (81.7%).

**Table 3:** Sociodemographic characteristics of study population by perceived academic performance.

Variable	Total	Perceived Academic Performance					*P*
		Good	Above average	Average	Below average	Poor	
Total (n)	35740	4900	9984	11060	6856	2940	
Gender (%)							
Male	51.49	50.7	53.7	53.8	49.5	41.1	<0.001
Female	48.51	49.3	46.3	46.2	50.5	58.9	
School type (%)							
Middle school	65.76	72.8	67.3	62.0	64.3	66.4	<0.001
High school	34.24	27.2	28.6	38.0	35.7	33.6	
Family structure (%)							
Nuclear	85.89	87.2	87.7	86.6	84.2	78.7	<0.001
Non-nuclear	14.11	12.8	12.3	13.4	15.8	21.3	
Maternal education (%)							
Elementary school and below	5.91	3.8	4.2	5.6	8.0	11.4	<0.001
Middle school	23.96	19.4	22.3	25.2	26.5	26.7	
High school	32.36	29.3	32.2	34.0	32.6	31.3	
Junior college	16.98	17.3	18.6	17.1	15.5	13.7	
Bachelor’s degree and above	20.80	30.2	22.7	18.0	17.4	16.9	
Extracurricular study time (%)							
<4 h/day	25.70	29.6	26.7	25.0	24.1	22.4	<0.001
≥4 h/day	74.30	70.4	73.3	75.0	75.9	77.6	

**Table 4:** Distribution of health-risk behaviors among study population (%).

Health-risk behaviors	Overall (N = 35740)
**Dietary behaviors**	
Did not eat breakfast on all 7 days	22.5
Did not drink milk on all 7 days	45.7
Drink soda ≥1 time/day	31.5
Eat dessert ≥1 time/day	20.6
**Physical activities and sedentary behaviors**	
Exercise ≥60 minutes for less than 5 days/week	65.4
Watch TV ≥3 hours/day	16.4
Surf the Internet ≥3 hours/day	15.7
Play video or computer games ≥3 hours/day	15.5
**Unintentional injuries**	
Jaywalking	43.2
Cycling violations	29.4
Swimming in unsafe regions	6.5
Severely injured	12.9
**Intentional injuries**	
Physical fight	18.3
Feeling unsafe on the way to and from school	27.7
Being bullied	48.3
Feeling lonely	61.7
Academic stress	81.7
Insomnia due to something	54.5
Feeling depressed	12.8
Runaway thoughts	28.5
Runaway behavior	6.9
**Substance abuse behaviors**	
Ever smoked	12.8
Currently smoked	2.4
Ever drank alcohol	54.4
Currently drank alcohol	21.7

### Association between health-risk behaviors and perceived academic performance

Table 5 reports the OR of the association between HRBs and students’ PAP adjusted by age, gender, school type, family structure, maternal education, extracurricular study time, and survey year.

**Table 5:** Association between exposure to health-risk behaviors and perceived academic performance in adolescents of three surveys.

Health-risk behaviors	Unadjusted		Adjusted^a^	
	OR (95% CI)	*P*	OR (95% CI)	*P*
**Dietary behaviors**				
Did not eat breakfast on all 7 days	0.69 (0.66–0.72)	<0.001	0.89 (0.86–0.93)	<0.001
Did not drink milk on all 7 days	0.78 (0.75–0.81)	<0.001	0.82 (0.79–0.85)	<0.001
Drink soda ≥1 time/day	0.97 (0.93–1.01)	0.098	0.98 (0.94–1.02)	0.244
Eat dessert ≥1 time/day	1.01 (0.97–1.06)	0.615	0.99 (0.95–1.04)	0.720
**Physical activities and sedentary behaviors**				
Exercise ≥60 minutes for less than 5 days/week	0.86 (0.83–0.90)	<0.001	0.87 (0.83–0.90)	<0.001
Watch TV ≥3 hours/day	0.91 (0.87–0.96)	0.001	0.93 (0.88–0.98)	0.007
Surf the Internet ≥3 hours/day	0.83 (0.78–0.89)	<0.001	0.87 (0.81–0.93)	<0.001
Play video or computer games ≥3 hours/day	0.87 (0.82–0.93)	<0.001	0.89 (0.84–0.95)	0.001
**Unintentional injuries**				
Jaywalking	0.96 (0.92–0.99)	0.024	0.96 (0.92–0.99)	0.033
Cycling violations	0.86 (0.83–0.90)	<0.001	0.91 (0.87–0.95)	<0.001
Swimming in unsafe regions	0.93 (0.87–1.01)	0.079	0.90 (0.84–0.97)	0.008
Severely injured	0.89 (0.84–0.94)	<0.001	0.89 (0.82–0.92)	<0.001
**Intentional injuries**				
Physical fight	0.86 (0.81–0.90)	<0.001	0.83 (0.79–0.88)	<0.001
Feeling unsafe on the way to and from school	0.98 (0.94–1.02)	0.350	0.97 (0.93–1.01)	0.153
Being bullied	0.89 (0.85–0.92)	<0.001	0.89 (0.86–0.93)	<0.001
Feeling lonely	1.10 (1.05–1.15)	<0.001	1.11 (1.06–1.16)	<0.001
Academic stress	0.80 (0.76–0.84)	<0.001	0.79 (0.75–0.84)	<0.001
Insomnia due to something	0.99 (0.95–1.03)	0.550	1.00 (0.96–1.05)	0.907
Feeling depressed	0.86 (0.82–0.92)	<0.001	0.87 (0.82–0.92)	<0.001
Runaway thoughts	0.86 (0.82–0.90)	<0.001	0.84 (0.80–0.88)	<0.001
Runaway behavior	0.79 (0.73–0.85)	<0.001	0.80 (0.74–0.86)	<0.001
**Substance abuse behaviors**				
Ever smoked	0.80 (0.75–0.85)	<0.001	0.85 (0.80–0.91)	<0.001
Currently smoked	0.93 (0.81–1.06)	0.280	0.97 (0.85–1.11)	0.688
Ever drank alcohol	1.01 (0.97–1.05)	0.574	0.99 (0.95–1.03)	0.748
Currently drank alcohol	0.98 (0.93–1.03)	0.342	0.98 (0.93–1.03)	0.419

^a^Adjusted for school, survey year, gender, age, maternal education, family structure and extracurricular study time.

#### Dietary behaviors

In the crude analyses, ate breakfast or drank milk on all 7 days was associated with PAP. Students who did not eat breakfast or drink milk on all 7 days tended to have a lower PAP, compared with students who ate breakfast or milk on all 7 days (*P*<0.001). After adjusting for all covariates, these association were also significant with the decreased odds of 0.89 (95%CI: 0.86–0.93, *P*<0.001) and 0.82 (95%CI: 0.79–0.85, *P*<0.001), respectively. While, drank soda or ate dessert ≥1 time/day was not associated with PAP.

#### Physical activity and sedentary behaviors

For physical activity, doing exercise ≥60 minutes for less than 5 days/week was associated with decreased PAP in the adjusted model (OR:0.87, 95%CI: 0.83–0.90, *P*<0.001). For sedentary behaviors, spending time on watching TV, or surfing the Internet or playing video game beyond 3 hours/day was at risk of a lower PAP with the adjusted decreased odds of 0.93 (95%CI: 0.88–0.98, *P* = 0.007), 0.87 (95%CI: 0.81–0.93, *P*<0.001), and 0.89 (95%CI: 0.84–0.95, *P* = 0.001), respectively.

#### Intentional injuries and unintentional injuries

We found that except for feeling unsafe on the way to school and insomnia, most of intentional injuries and unintentional injuries were associated with PAP. The effect was stronger for academic stress than other behaviors, with the adjusted decreased odds of 0.79 (95%CI: 0.75–0.84, *P*<0.001). On the other hand, feeling lonely in the past 12 months was positively associated with PAP, with an increased odds of 1.11 (95%CI:1.06–1.16, *P*<0.001) in the adjusted model.

#### Substance abuse behaviors

After adjustment, results showed that students who ever smoked as compared to their counterparts were less likely to have a higher PAP (OR:0.85, 95%CI:0.80–0.91, *P*<0.001). Other substance abuse behaviors were not found to be associated with PAP.

## Discussion

The present study examined the association between student’s PAP and HRBs involved in diet, physical activity and sedentariness, intentional and unintentional injuries, and substance abuse, with a sample of 12–18 year old Chinese adolescents in Shanghai. We found that students who had HRBs were more likely to present a lower PAP, compared to those who adopted health behaviors.

The results indicated that students who had not eaten breakfast on all days per week was a risk factor for lower PAP. It is consistent with previous systematic review demonstrating that habitual breakfast pattern is positively correlated with students’ academic performance [25]. A UK study reported that academic performance decreased with lower frequencies of school-day breakfast consumption in adolescents [26]. Breakfast was believed to be the most important meal of the day, which contributed to 25%-30% of total daily energy [27]. With the increase in breakfast frequency, energy level throughout the day and levels of protein, calcium, iron, zinc, selenium, retinol, and other nutrients consumed significantly increased. Some of these nutrients play an important role in brain development and cognitive levels of learning [28]. Those who skipped breakfast might have lower blood sugar levels, resulting in reduced cortical excitability and difficulty concentrating [29]. On the other hand, studies have shown that motivation to learn mediates the relationship between breakfast consumption and academic performance [30]. Thus, students who do not eat breakfast might decrease alertness and motivation in class, and thereby affect their performance.

We also found that students who participated in long-term physical activities or spent shorter hours on screen media are apt to get good PAP, compared to their counterparts. Several reviews have shown that physical activities improve the academic performance of children and adolescents. A study showed that the relationship between physical activity and academic performance was mediated through cognition—that is, physical activity promoted cognitive development in children, showing a positive impact on academic performance [31]. A Japanese study also showed that children who participated in more physical activities and spent less time looking at screens had better academic performance than children who were less engaged in less physical activities and more engaged with screens [32]. A study conducted on Chinese adolescents reported that spending more time on television, videos, and social networking sites were negatively associated with academic performance [18]. Screen exposure (especially TV-viewing) is a passive behavior required little intellectual input. The prolonged video screen use may stimulate impulsive behavior and cause attention deficits, adversely affecting children’s intellectual development and cognition [33].

Unintentional injury is a risk factor of lower PAP. It always accompanied with sickness absenteeism and disability. An India cross-sectional study shown that the sickness absenteeism of unintentional injuries averaged over one week, and about half of injured children missed school after an injury [34]. Students who are more frequently absent from school have missed teacher-led lessons, peer interactions, and other learning activities, and are consequently likely to obtain lower academic performance [35–37]. With regard to intentional injury, we found that psychological health problems including internalizing and externalizing behaviors were negatively associated with academic performance. These findings are consistent with other studies, for example, symptoms of depression have negative effect on students’ attention and memory [38, 39]. A study in the UK reported that depression predicted a decline in examination performance [40]. Moreover, studies aimed at undergraduate students supports that academic stress is an impediment to academic performance, [41–43] although there were few study focused on the association between academic stress with academic performance in adolescents. A moderate level of academic stress helps to increase academic self-efficacy and obtain better educational outcomes [42]. However, once students experienced high stress levels beyond their load-bearing potential, it could result in school burnout and worse performance [43]. Regarding externalizing behaviors (physical fight and bullying), several studies are consistent with our findings. A study conducted on American high school students showed that a low self-perception of academic performance was associated with being involved in a physical fight [44]. A Spanish study reported that students in the role of the bully/victim had lower academic performance, compared with those in non-bully/non-victim [45]. Bullying has a dual direction, such that being bullied may lead to future aggressive behaviors [46]. Exposure to physical fight or bullying situations affected students’ perceptions of safety and belonging at school, harmed social relationships, and caused difficulties in academic perform and dropping out of school [47, 48]. For substance abuse behaviors, our results indicated that students who had ever tried cigarette smoking tend to present lower academic performance. Animal studies have shown that nicotine has effects on deficits in cognitive function and emotional dysregulation in adolescent mice [49]. In addition, students with poor academic performance are more susceptible to cigarette smoking [50]. A study in South Korea found that students with poor academic performance are at higher risk of smoking than those with good academic performance [51]. Therefore, smoking is likely to push students into a vicious cycle of poor academic performance.

The main strength of our study is the first large population-based study that has examined possible association of HRBs with PAP through a well-designed survey among adolescent students in Shanghai, China. In addition, the investigation contained comprehensive domains of HRBs. This study also has several limitations. Firstly, this was a cross-sectional study, with the data from a given time point, which cannot determine the causal relationship (if any) between HRBs and academic performance among middle and high school students. Secondly, the collected data from self-reported questionnaires tend to be subjective, although the questionnaires were generally confirmed to be reliable in the test-retest reliability analysis. The presence of recall bias also should not be ignored. Finally, this study only analyzed the association between each HRB and PAP. Further study is needed to focused on clustering and co-occurrence of HRBs among adolescents.

### Public health implication

The results of this study suggest that, in addition to students’ academic performance, parents and educators in schools should concern about the multiple HRBs in adolescents. Based on these findings, we recommend effective interventions during adolescence. It is an opportunity to rectify health problems that have arisen during childhood, form a healthy lifestyle, and prevent adolescents from undermining health in adulthood [11]. We should strengthen the collaboration between schools, families, and communities to push forward health promotion programs directed toward HRBs including but not limited to promoting regular breakfast intake projects, improving students’ nutritional status, enriching extracurricular activities, and managing screen time. In addition, schools and families should promote health education to increase the students’ awareness of unintentional injuries, and pay more attention to mental status of students to avoid intentional injuries.

## Conclusions

In conclusion, HRBs were significantly associated with PAP among adolescents in Shanghai. Students who had irregular breakfast, spent less time on physical activity and more time on exposure to the screen, and experienced intentional/non-intentional injuries were more likely to have a lower academic performance. Our findings highlight the requirement to pay special attention to the students who had multiple HRBs.
